# Functional Geometry of Human Connectomes

**DOI:** 10.1038/s41598-019-48568-5

**Published:** 2019-08-19

**Authors:** Bosiljka Tadić, Miroslav Andjelković, Roderick Melnik

**Affiliations:** 1 Department of Theoretical Physics, Jožef Stefan Institute, 1000 Ljubljana, Slovenia; 2 Complexity Science Hub, Josefstaedter Strasse 39, Vienna, Austria; 3 Institute of Nuclear Sciences Vinča, University of Belgrade, 1100 Belgrade, Serbia; 4 MS2Discovery Interdisciplinary Research Institute, M2NeT Laboratory and Department of Mathematics, Wilfrid Laurier University, 75 University Ave W, Waterloo, ON N2L 3C5 Canada; 5 BCAM - Basque Center for Applied Mathematics, Alameda de Mazarredo 14, E-48009 Bilbao, Spain

**Keywords:** Applied mathematics, Scientific data

## Abstract

Mapping the brain imaging data to networks, where nodes represent anatomical brain regions and edges indicate the occurrence of fiber tracts between them, has enabled an objective graph-theoretic analysis of human connectomes. However, the latent structure on higher-order interactions remains unexplored, where many brain regions act in synergy to perform complex functions. Here we use the simplicial complexes description of human connectome, where the shared simplexes encode higher-order relationships between groups of nodes. We study consensus connectome of 100 female (F-connectome) and of 100 male (M-connectome) subjects that we generated from the Budapest Reference Connectome Server v3.0 based on data from the Human Connectome Project. Our analysis reveals that the functional geometry of the common F&M-connectome coincides with the M-connectome and is characterized by a complex architecture of simplexes to the 14th order, which is built in six anatomical communities, and linked by short cycles. The F-connectome has additional edges that involve different brain regions, thereby increasing the size of simplexes and introducing new cycles. Both connectomes contain characteristic subjacent graphs that make them 3/2-hyperbolic. These results shed new light on the functional architecture of the brain, suggesting that insightful differences among connectomes are hidden in their higher-order connectivity.

## Introduction

Human psychology and behaviour are determined by functional brain connectivity among neurons, neural assemblies, or entire regions, making the patterns of circuitry that can be detected by brain imaging^1^. Recent large-scale research into the brain imaging data within the Human Connectome Project (HCP)^2–4^ aims to uncover, describe and understand the functional structure of human connectome; the connectome is visualised as a network consisting of different brain regions (grey matter) and paths between them (white-matter fibre bundles) that can be determined by mapping the diffusion-MRI and tractography data. The network nodes are identified as distinct brain regions that are functionally similar and spatially close as well as equally connected to the other regions^4–7^. The connections between these regions are inferred from brain imaging data. Recent studies provided insight into the developmental trajectory, elucidating that the architecture of connections in the brain develops over time to support the function^8^. Thus the inferred structure of edges may vary among different subjects, performed tasks and conditions. In this context, the sex-related differences in brain connectivity evolve across the development to accompany all functional and behavioural dimensions^8,9^. Therefore, the consensus between the pipelines in the structural connectome can be mapped from a large population tractography data^10^ and depends on many parameters. Based on the data from HCP^2^ and the brain mapper developed in^11^, the Budapest connectome server^12^ provides the possibilities to infer the *consensus networks* at a variety of the relevant parameters, as described in^13^. The mapping of imaging data to the brain networks enables an objective analysis based on graph theory methods^14,15^.

Recently, different studies of brain imaging data revealed the strong evidences for sex-related differences in the structural connectome^16–22^. This subject was not well researched, but already it brought some controversial debates^9^. The exact origin of these differences and their potentials and impact on the level of individual and social behaviour are still to be investigated^23^. On the other hand, the current degree of reliability of the connectome data provides an opportunity for a mathematical analysis of structural differences at all levels. For example, a recent study^22^ has shown that the consensus female connectome has superior connectivity than the consensus male connectome in many graph-theoretic measures.

Recent investigations of geometrical properties of various complex systems^24–33^ show the relevance of the higher-order connectivity beyond standardly considered pairways interactions. Mathematically, the impact of these higher order interactions is adequately described by the simplicial complexes in the algebraic topology of graphs^34–37^. In these complexes, elementary geometrical shapes (triangles, tetrahedra, and simplexes of higher order) are combined through shared substructures of various orders. These geometrical structures directly influence dynamic processes that the complex system in question performs, such as transport, diffusion, or synchronisation among the involved nodes. In the case of brain networks, the main dynamic function pertains to maintaining an optimal balance between the processes of integration and segregation where different regions of the brain can be simultaneously involved and the present modular structure of the brain plays an important role^38–41^. Anatomical modules of the brain, which are recognized as different mesoscopic communities in the network^42–45^, are based on spatial topography and coexpression of genes in the brain cells^46^. It has been suggested that each module performs a discrete cognitive function while specific connector nodes take on communication between modules^40^. However, the fine functional organisation inside these modules remains unexplored. Besides, the occurrence of simplicial complexes causes the emergent hyperbolicity or a negative curvature^47^ in the structure of the graph, which affects its functional properties. In this sense, the complete graph and associated tree are ideally hyperbolic, characterised by the hyperbolicity parameter $\delta =0$. The graphs with small values of *δ* are subject to intensive investigations for their ubiquity in natural and social systems, as well as in technology applications^24,25,30,33,48^. Moreover, current theoretical studies reveal that Gromov hyperbolic graphs with a small hyperbolicity parameter have specific mathematical properties^48^. In particular, the bounds for the *δ*-parameter of the whole graph can be derived from subjacent simpler graphs, for example, induced cycles or clique separators of a given length^49–54^. Therefore, the study of the hyperbolicity of brain graphs can reveal the presence of typical local structures that are potentially decomposable into some known forms, which underlie the brain’s dynamic complexity.

In this work, we considerably expand the analysis of human connectome beyond the simple pairwise connectivity. Using the mathematical techniques of algebraic topology of graphs, we identify hierarchically organised complexes that encode higher-order relationships between regions of the brain and explore the hyperbolic geometry of brain graphs. We consider the consensus connectomes mapped from 100 female (F-connectome) and 100 male (M-connectome) subjects, using the brain mapper and imaging data from the Human Connectome Project, which is provided by the Budapest server 3.0^12^. The weighted edges are inferred according to the *electrical connectivity* criteria, which are most sensitive to the number of fibres observed in the tractography data. We analyse the connectomes that correspond to the significant variation in the number of fibres launched (see Methods). With the appropriate topology measures, our objectives are to determine the hidden structure of human connectome endowed with the relationships between groups of nodes and express the possible gender differences in this context. To this end, we construct and investigate a common F&M-connectome at different numbers of fibres and determine its structure, parametrised by simplicial complexes, and the graph’s hyperbolicity parameter. Furthermore, by comparing edges in the F- and M-connectomes, we identify the excess edges that appear consistently in the F-connectome with an increased number of fibres. Our mathematical analysis reveals a rich structure of simplicial complexes that are common to the F&M-connectome and belong to different brain anatomical communities and cycles that connect them inside and across the two brain hemispheres. It further confirms the higher connectivity of the F-connectome and demonstrates that the excess edges have a well-organised structure that includes a particular set of paths and brain regions.

## Methods

### Input data & consensus networks

The consensus connectomes that we study are generated at the Budapest Reference Connectome Server v3.0^12^ using data from the Human Connectome Project (HCP) for 500 individuals^2^. As it is described in^13^, the server produces the connectomes for each anonymised individual based on its diffusion MRI data of HCP and by applying the brain mapping tools^11,55,56^ for parcellation, tractography, and graph construction. From these individual graphs, the consensus connectome with the edges that are common for a specified group of individuals is generated, corresponding to the settings of a variety of parameters^13^. Apart from the resolution, the number and biological sex of individuals can be selected, the method for computing the weights of edges, as well as three options regarding the number of fibres launched in the tractography phase. For our study, we have selected the data that provide the *consensus networks* for female connectome and male connectome based on 100 subjects of each sex. The corresponding brain networks consist of N = 1015 nodes (anatomically annotated brain regions) and the weights of the connections between them determined according to the *electrical connectivity* criteria, i.e., the number of fibres between the considered pair of regions is divided by the average fibre length. We consider three different fibre counts, comprising of *N*_*F*_ = 20 K, 200 K, and 1000 K fibres, where for short K ≡ 1000. For the additional parameters, we have set the minimum edge confidence as 100%, minimum edge weight as 4, and the median weight calculation. The resulting adjacency matrices of the weighted networks, herewith called F-connectome and M-connectome, respectively, are downloaded together with the node labels, i.e., the names of the anatomical brain regions.

### Gromov hyperbolicity parameter of graphs

A generalization of the Gromov notion of hyperbolicity^47^ is applied to graphs endowed with the shortest-path metric. Specifically, the 4-point Gromov criterion states that a graph *G* is *δ*-hyperbolic *iff* for any four vertices $(A,B,C,D)$ there is a fixed small value *δ*(*G*) such that the following relation beween the sums of distances ${\mathscr{S}}$ ≡ *d*$(A,B)+d(C,D)\le  {\mathcal M}$ ≡ $d(A,C)+d(B,D)\le  {\mathcal L}$ ≡ $d(A,D)+d(B,C)$ implies $d(A,D)+d(B,C)-d(A,C)-d(B,D)\le 2\delta (G)$. Thus, for a *δ*-hyperbolic graph, there is *δ*(*G*) such that any four nodes of the graph satisfy the condition

1 $$\delta (A,B,C,D)\equiv \frac{ {\mathcal L} - {\mathcal M} }{2}\le \delta (G).$$

From the triangle inequality, the value of $( {\mathcal L} - {\mathcal M} )/2$ is bounded brom above by the minimal distance ${d}_{min}\equiv min\{d(A,B),d(C,D)\}$ in the smallest sum ${\mathscr{S}}$. This relationship enables a direct computation of the hyperbolicity parameter of a graph, which is given by its adjacency matrix. In particular, by sampling a large number (10^9^) 4-tuples of vertices we plot $\delta (A,B,C,D)$ against the corresponding *d*_*min*_; the plot saturates at larger distances. We compute the average $\langle \delta \rangle$ for all *d*_*min*_ as well as ${\delta }_{max}=ma{x}_{G}\{\delta (A,B,C,D)\}$ as the largest value observed in the entire graph, which gives *δ*(*G*).

We also determine the distribution *P*(*d*) of the shortest-path distances *d* on the graph. The largest distance defines the graph’s *diameter D*, which gives the upper bound to the hyperbolicity parameter, $\delta (G)\le D\mathrm{/2}$. As mentioned above, the hyperbolic graphs with a small parameter *δ* have a specific structure of subgraphs, from which the upper bound of *δ*(*G*) can be derived^50–54^. In this context, the following definitions apply. A subgraph ${\rm{\Gamma }}$ of *G* is called *isometric* if the distance between every pair of vertices $(A,B)\in {\rm{\Gamma }}$ is equal to the distance between them measured on *G*, i.e., ${d}_{{\rm{\Gamma }}}(A,B)={d}_{G}(A,B)$. A *cycle C*_*n*_ is a sequence of *n* pairwise connected vertices with $n+1\to 1$; an *induced cycle* does not contain a *chord*, an edge connecting nonconsecutive vertices. A *clique* of size $s\equiv {q}_{max}+1$ is the full graph of *s* vertices and $s(s-\mathrm{1)/2}$ edges.

### Q-analysis of graphs: definition of structure vectors

Considering a connectome as an undirected and unweighted graph *G*, the higher-order connectivity of its vertices can be appropriately parametrised by the maximal complete subgraphs (or cliques) whose vertices belong to a clique complex *C*(*G*) in the graph *G*^36^. Two cliques *σ*_*r*_ and *σ*_*q*_ of the orders *r*, *q* can be interconnected by sharing some vertices; then the structure made by the shared vertices represents a common face of both cliques. For example, if for $r < q$ all vertices of *σ*_*r*_ belong to *σ*_*q*_, then the simplex *σ*_*r*_ represents a face of the order *r* in the simplex *σ*_*q*_. The simplicial complex represents the aggregate of cliques that share the faces of different orders $q=0,1,2\cdots {q^{\prime} }_{{\max }}-1$, where ${q^{\prime} }_{{\max }}$ indicates the order of the largest clique in the complex. The order of a simplicial complex is the largest order of a simplex in it; we denote by *q*_*max*_ the order of the largest complex in the entire graph.

Applying the Bron-Kerbosch algorithm^57^, the adjacency matrix of the graph *G* is converted into the incidence matrix Λ, which contains all cliques in the graph by identifying the vertices that belong to them; using this information, we then find how different cliques interconnect via shared nodes to make the higher-order structures. The overall hierarchical organisation of the graph can be quantified^26,27,58–61^ by three *structure vectors* having the components along different topology levels $q=1,2,3,\cdots {q}_{{\max }}$. Specifically, for each considered graph, we determine:

- FSV—the first structure vector $\{{Q}_{q}\}$, where each *Q*_*q*_ represents the number of *q*-connected components;
- SSV—the second structure vector $\{{n}_{q}\}$, where *n*_*q*_ indicates the number of connected components from the level *q* upwards;
- TSV—the third structure vector $\{{\hat{Q}}_{q}\}$ is introduced to quantify the degree of interconnectivity between cliques at each level *q*, and can be derived from the other two as ${\hat{Q}}_{q}=1-{Q}_{q}/{n}_{q}$.

These structure vectors provide a measure of the graph’s global architecture (see^62^ for the application of *Q*-analysis for the vertex neighbourhood). Note that, in this context, the 1-skeleton of the simplicial complex consists of nodes and edges, representing the topological graph, which is analysed by graph theory methods. We also determine some graph measures^63,64^ and community structure^43,65,66^ of the corresponding topological graphs, see Results. *Visualisation and standard graph parameters* are made by using Gephi software^67^.

## Results

### Consensus networks of human connectome

**Figure 1:**
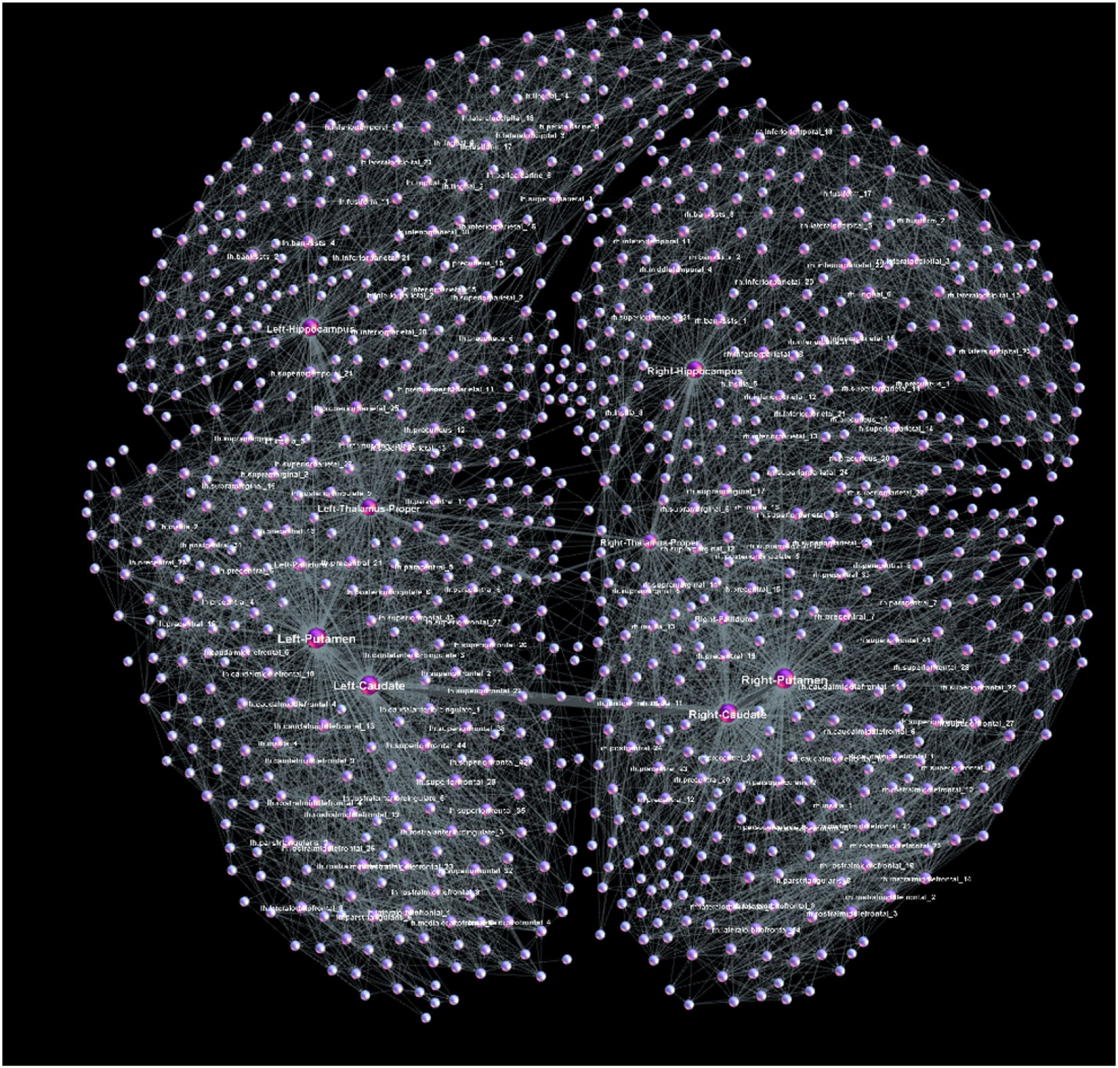
The female connectome at the highest resolution consisting of 1115 nodes (brain regions) and 11339 edges between them. The network is deduced from the HCP data provided at the server^12^ with weighted edges as the median for 100 female subjects and *N*_*F*_ = 1000 K fibres launched between each pair of nodes.

**Figure 2:**
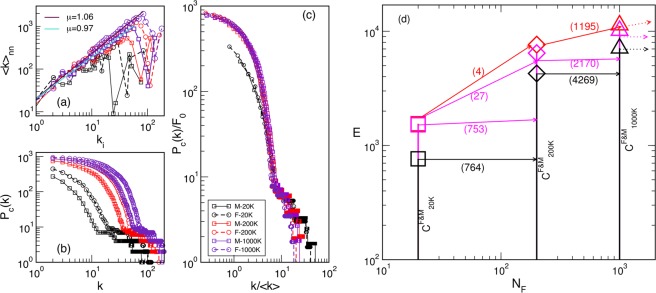
Assortative correlations among the degree of the neighbouring nodes (**a**), the degree distributions (**b**) and the scaled degree distributions (**c**) for the consensus female F- and male M-connectome at the different number of fibres *N*_*F*_, as the legend indicates. (**d**) Schematic view of the number of edges *E* and their co-occurrence in the connectomes at the increasing number of fibres *N*_*F*_, see also Table 1. The common ${C}^{F\& M}$-connectome at a large *N*_*F*_, inherits all edges from the ${C}^{F\& M}$ at a lower *N*_*F*_, black lines, plus a fraction of the excess edges of the F-connectome, shown by pink lines. The top line (red) shows the number of robust excess edges in F-connectome which do not appear in any of the common ${C}^{F\& M}$-connectomes at a larger *N*_*F*_.

According to the parameter settings (see Methods), the considered F-connectome consists of the edges that appear in all 100 female subjects, and similarly, the M-connectome contains the edges that are present in all 100 male subjects. For the illustration, the F-connectome at 1000 K fibres is shown in Fig. 1 with the labelled brain regions as nodes. Here, we use the simplicial complexes parametrisation (see Methods) and the graph’s hyperbolicity measures to uncover the hidden structure of human connectome, which is encoded in the higher-order connectivity between groups of nodes. Furthermore, using these mathematical measures, we analyse the variations of the brain connectivity patterns in female and male connectomes, depending on the number of fibres *N*_*F*_ launched in the tractography. As it is shown in Fig. 2a–d, the number of connections increases with *N*_*F*_ and differs between F- and M-connectomes, resulting in different degree distributions, cf. Fig. 2b. However, these distributions fall to the same curve when plotted against the rescaled node’s connectivity $k/\langle k\rangle$, as shown in Fig. 2c. In the node’s first neighbourhood, the average connectivity correlates with the node’s degree *k*_*i*_ in an *assortative* manner i.e., ${\langle k\rangle }_{nn}\sim {k}_{i}^{\mu }$ with a positive exponent *μ*, cf. Fig. 2a, with the outlier nodes representing big hubs (“rich club” organisation^14^). The scaling range and the exponent *μ* are slightly larger in the F-connectome. We show in the following that a significant difference between these connectomes lies in the structure of simplicial complexes.

**Figure 3:**
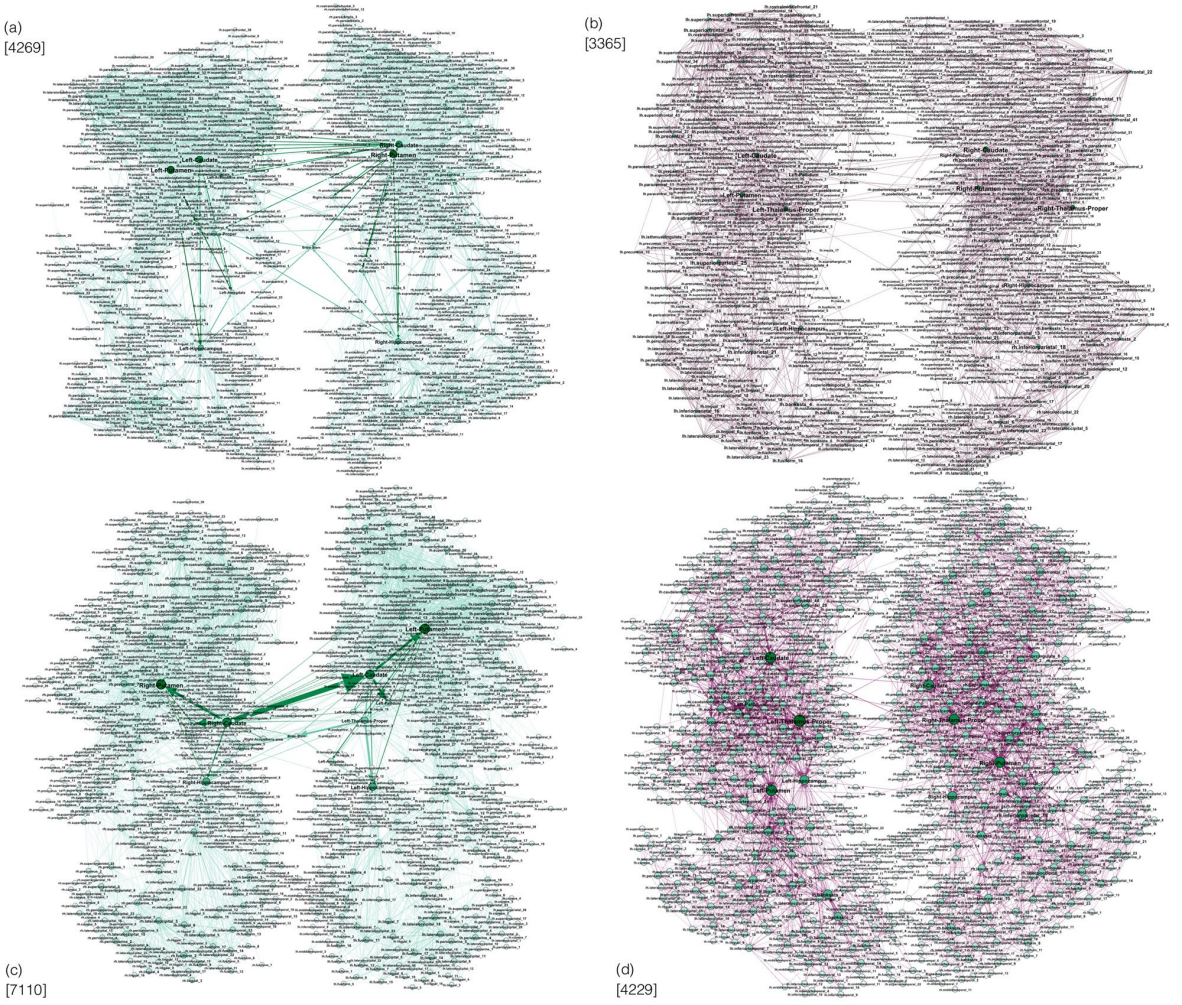
Networks of connections established among labelled brain regions at different numbers of launched fibres *N*_*F*_: (**a**) Common M&F connectome at ${N}_{F}=200\,K$ and (**c**) common M&F connectome at ${N}_{F}=1000\,K$, the weights of M-connectome are shown. (**b**,**d**) The patterns of the additional edges appearing in the F-connectome (F-excess), which are not present in the M-connectome at *N*_*F*_ = 200 K and ${N}_{F}=1000\,K$, respectively. The numbers of edges in the corresponding graph are indicated at each figure. The number of edges is inherited in the target graph at ${N}_{F}=1000\,K$ from the graphs at ${N}_{F}=200\,K$. Explicitly, the graph (**c**) inherits all edges from the graph (**a**). The 2170 edges from the graph (**b**) appear in the common connectome (**c**), whereas 1195 edges of the graph (**b**) are inherited as the excess edges in the graph (**d**).

To proceed, we first identify all edges that (although with different weights) are common for both F-connectome and M-connectome, here called ${C}^{F\& M}$-connectome at different *N*_*F*_. Table 1 and Fig. 2 summarise the number of edges and mutual relationships of different connectomes. Figure 3 shows the corresponding graphs with the labelled brain regions, obtained for *N*_*F*_ = 200 K and 1000 K. Specifically, we find that:

- The number of established edges in each considered connectome increases with the number of fibres launched *N*_*F*_;
- The common ${C}^{F\& M}$-connectome practically coincides with the M-connectome at each *N*_*F*_, whereas the F-connectome contains an increasing number of excess edges with the increasing *N*_*F*_;
- The common ${C}^{F\& M}$-connectome at a higher *N*_*F*_ inherits all edges from the ${C}^{F\& M}$-connectome at a lower *N*_*F*_;
- A significant fraction of the excess edges found in the F-connectome at a lower *N*_*F*_ appear in the common ${C}^{F\& M}$-connectome but at a higher *N*_*F*_;
- There is a large number of the excess edges in the F-connectome that are never found in the common ${C}^{F\& M}$-connectome at a higher *N*_*F*_; the patterns of these edges make the fundamental difference between the human female and male connectomes.

**Table 1:** For the number of launched fibres *N*_*F*_, the corresponding number of edges are shown in the consensus male (M) and female (F) connectomes, the edges ${C}^{F\& M}$ common to F&M connectomes, and the total number *F*^*e*0^ of excess edges in the F-connectome; the fractions of *F*^*e*0^ indicated as *F*^*ec*+^ and *F*^*ecc*+^ are the edges that appear in the common connectomes at the two higher *N*_*F*_, respectively, while *F*^*ex*^ are the excess edges also at the higher *N*_*F*_.

*N*_*F*_	M	F	*C* ^*F*& *M*^	*F* ^*e*0^	*F* ^*ec*+^	*F* ^*ecc*+^	*F* ^*ex*^
20000	776	1548	764	784	753	27	4
200000	4285	7634	4269	3365	2170	—	1195
1000000	7110	11339	7110	4229	—	—	≥1195

The difference between *M* and *C*^*F*&*M*^ at 20 K and 200 K consists of 12 and 16 edges, which all appear in *C*^*F*&*M*^ at 1000 K.

### The structure of simplicial complexes in brain graphs

According to Table 1 and Fig. 2, at each *N*_*F*_, the common *F*&*M*-connectome practically coincides with the male connectome (apart from the exact weights of edges) while there are many excess edges in the female connectome. Here, by applying *Q*-analysis (see Methods) to the corresponding graphs at different numbers of fibres *N*_*F*_, we show that (i) the common human connectome possesses a nontrivial hidden structure encoding multi-vertex connectivity; (ii) the excess edges of the F-connectome are not random but exhibit a highly organised structure, which thus implies a specific functionality, cf. Fig. 3.

**Figure 4:**
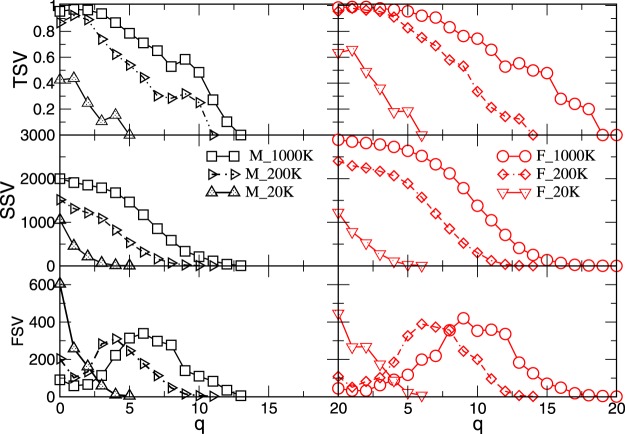
The components of three structure vectors defined in Methods (FSV,SSV, TSV) plotted against the topology level *q* for the consensus connectomes determined from 100 male (left) and 100 female (right) subjects with the varied number of fibres *N*_*F*_, indicated in the legend.

In Fig. 4 the results for the three structure vectors, defined in Methods, are presented for different *N*_*F*_. As Fig. 4 shows, the structure of connectomes becomes richer with the increased number of fibers *N*_*F*_. In particular, the cliques of a systematically larger order *q* appear and the degree of their inter-connectivity increases as measured by TSV. Moreover, the larger number of edges in the F-connectome leads to a much richer structure of the simplicial complexes, which is expressed by all structure vectors, cf. right panels of Fig. 4. We also notice that the difference between the M- and F-connectomes systematically increases with the increased *N*_*F*_. Representative quantitative properties are given in Tables SI-I and SI-II in Supplementary Information. Noticeably, the ${Q}_{q=0}$ component of the FSV, which gives the number of fragments of the graph, suggests that besides the largest component some vertices and small clusters remain disconnected. The number of fragments decreases and the connectivity increases with the increasing *N*_*F*_. The corresponding number of edges in the largest cluster is given in Table 1. The organisation of the present edges at each *N*_*F*_ manifests in the presence of simplicial complexes with the largest order *q*_*max*_. From Fig. 4 and Table SI-II, we see that the F-connectome possesses the cliques of a higher order; the difference increases from ${q}_{max}^{M}=5$ and ${q}_{max}^{F}=6$, at 20 K, to ${q}_{max}^{M}=13$ and ${q}_{max}^{F}=20$, at 1000 K. The number of cliques of the highest order is different, as well as their connection to the other cliques at the level just below the *q*_*max*_. Apart from the increased number of topology levels, the F-connectome also exhibits a significant degree of interconnections between the big cliques. For example, the TSV for the F-connectome at the level *q* = 13, which equals to ${q}_{max}^{M}$, is still very high, about 55%. Below, we identify the excess edges in the F-connectome and examine the patterns which they make. We note that the presence of larger simplicial complexes and their better inter-connections in the F-connectome, as compared to the M-connectome, agree well with the observed differences in some non-local graph properties, for example, the degree-related correlations, cf. Fig. 2a. Similarly, this structure of simplicial complexes can be compatible with the better expander graph and the width of balanced cuts in the female connectomes, reported in^22^ by graph-theory analysis. Meanwhile, the dissimilarity in the node’s degree distributions can be scaled out. Hence, the insightful differences among these connectomes are in the architecture of connections, not merely in their number. For completeness, in Table 2 we show a summary of different graphs’ properties.

**Table 2:** Summary of graph parameters for the F-connectome and the M-connectome (which is equivalent to the common F&M-connectome) and the excess edges (F-excess) in the F-connectome at 1000 K.

Graph	〈*k*〉	〈${\boldsymbol{\ell }}$〉	〈*Cc*〉	*ρ*	*mod*	*D*	*δ*_*max*_	*q*_*max*_
F-conn (Fig. 1)	12.07	3.45	0.69	0.025	0.59 (6)	8	3/2	20 (1)
M-conn (Fig. 6a)	7.01	3.97	0.67	0.014	0.62 (6)	8	3/2	13 (6)
F-excess (Fig. 3d)	4.17	4.36	0.13	0.008	0.654	11	5/2	3 (149)
F-excess1195 (Fig. SI-3)	1.77	5.91	0.064	0.005	0.689	17	4	2 (112)
F-excess1195w18 (Fig. 6b)	1.41	6.54	0.031	0.008	0.764	19	4	2 (18)
randomised-F-excess1195	0.94	9.95	0.006	0.003	0.898	30	5	2 (1)

The parameters of the F-excess1195 and its subgraph with large weights of edges F-ex1195w18, as well as its randomised version are shown. The quantities are computed for undirected graphs: the average degree 〈*k*〉, path length 〈$\ell$〉 and clustering coefficient 〈*Cc*〉, the graph’s density *ρ*, modularity *mod* and (the number of communities), diameter *D*, hyperbolicity parameter *δ*_*max*_, and the highest topology level *q*_*max*_ with the number (*Q*_*q*_) of the simplexes of that order.

### Hyperbolicity of the human connectome

**Figure 5:**
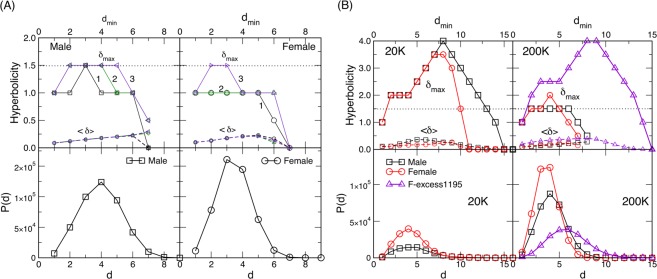
(**A**) Hyperbolicity parameters *δ*_*max*_ (upper curves, full lines) and $\langle \delta \rangle$ (lower curves, dashed lines) plotted against *d*_*min*_ for the female and male consensus connectomes for *N*_*F*_ = 1000 K fibres launched. Three lines 1,2 and 3 are for 10^7^, 10^8^ and 10^9^ sampled 4-tuples, respectively. Bottom row: The distribution *P*(*d*) of the shortest-path distances *d* for the corresponding female and male connectomes. (**B**) Hyperbolicity parameters *δ*_*max*_ and $\langle \delta \rangle$ (top panels) and the shortest-path distances distribution (bottom panels) of the consensus female and male connectomes for the numbers of fibres *N*_*F*_ = 20 K and 200 K indicated on the panel, and for the excess edges in the female connectome at 200 K, described in the text as F-excess1195. The number of sampled 4-tuples is 10^9^.

Another hidden geometrical property of the connectomes is their hyperbolicity in the graph-metric space, as we show in the following. A previous study^68^ refers to the brain network embedded in a hyperbolic space, i.e., the volume of the skull. Theoretically, the hyperbolicity of a path-connected geodesic metric space was proved^69,70^ to be equivalent to the hyperbolicity of the graph associated with it. In the brain graphs studied above, the hierarchical organisation of simplicial complexes reduces the distances between nodes in the graph’s metric space, which implies their hyperbolicity. Here, using the 4-point Gromov criterion (see Methods), the hyperbolicity parameters are determined for F- and M-connectomes obtained by varying the number of fibres *N*_*F*_. In this context, we consider the corresponding adjacency matrix of the largest connected cluster as an unweighted symmetrical graph. Figure 5A shows the results for the largest available ${N}_{F}=1000\,K$. In the bottom panels, the histograms of the distances between all pairs of vertices are plotted. Although the diameter *D* = 8 applies to both graphs, typical distances in the F-connectome appear to be smaller. In the top panels, we plot the values of the *δ*-parameter against the minimum distance *d*_*min*_ of a given 4-tuple, as described in Methods. Specifically, lower sets of curves represent the average value $\langle \delta \rangle$ for a given *d*_*min*_. Whereas the top lines contain the recorded maximum value *δ*_*max*_ from all considered 4-tuples.

We observe that the values of $\langle \delta \rangle$ are very low, practically never exceed 0.25, which suggests the impact of the types of local structures populated by cliques. They are 0-hyperbolic subgraphs (atoms)^52^ and induced cycles, whose hyperbolicity depends on the length of the cycle and can be expressed as a multiple of 1/4^48^. Moreover, ${\delta }_{max}=\mathrm{3/2}$ suggests that dominant isometric subgraphs, which determine the value of *δ*_*max*_ for the whole graph^54^ in both connectomes, can be cycles *C*_*n*_ that have $n\ge 6$ but with the diameter $D\ge 3$. While we regularly obtain ${\delta }_{max}=\mathrm{3/2}$ in the M-connectome, it was necessary to sample 10^9^ different 4-tuples to find it in the F-connectome. Meanwhile, the value of ${\delta }_{max}=1$ occurs often in the F-connectome. It suggests that the dominant subgraphs can be composed of cliques that are one-edge apart, which, according to the results in^32,52^, yields that ${\delta }_{max}={\delta }_{clique}+1$ or they contain short cycles isomorphic to 4-cycle^48^. The situation is considerably different at the lower number of fibres where both F- and M-connectomes have gradually fewer edges (see Table 1). Consequently, the distances between vertices increase as well as the diameters of the graphs. The increased distances lead to the appearance of larger cycles and yield the distortion of the hyperbolicity parameter^51^ while the graphs remain hyperbolic; we find the upper bound ${\delta }_{max}\le 4$ in both connectomes, as shown in Fig. 5B.

### The structure of common F&M-connectome and the excess edges in Female connectome

**Figure 6:**
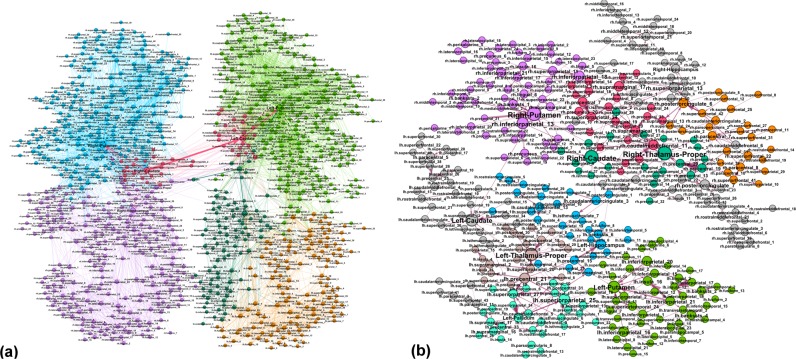
(**a**) The common F&M-connectome at ${N}_{F}=1000\,K$ with labelled brain regions belonging to the brain anatomical communities, indicated by different colours. Weights of the edges are from the M-connectome. (**b**) The robust structure of the excess connections among brain regions (labels) in the consensus female connectome that cannot be found in the consensus male connectome with up to 1000 K fibres launched. Different colours indicate weighted communities. We show only the 490 edges with the significant weight in the tale of the weight distribution, cf. Fig. SI-1, and the involved 348 brain regions.

By performing the edge-by-edge comparisons in the corresponding graphs, see Fig. 3, we identify every edge in terms of its source and destination vertex and the weight. For the highest *N*_*F*_, the common *F*&*M*-connectome consists of 7110 edges which coincide with the structure of the M-connectome, cf. Table 1 and Fig. 2. The corresponding network of the M-connectome, as shown in Fig. 6a, possesses a characteristic community structure related to different anatomical brain regions. Apart from the heterogeneity of the structure due to different degrees and weights of edges, this community structure is essential for the brain functional complexity^39–45^ for both F- and M-connectomes. As mentioned above, the F-connectome possesses an extra structure on the top of the common F&M-connectome; it consists of many edges that connect different brain regions. The number of the extra edges varies with the number of launched fibres *N*_*F*_, as shown in Table 1. A subgraph of the identified excess edges in the F-connectome, here termed *F-excess1195*, consists of 1195 edges which systematically appear in the F-connectome, first at ${N}_{F}=200\,K$ and then at ${N}_{F}=1000\,K$ with increased weights; these edges are not present in the corresponding M-connectomes, and thus are not part of the universal *F*&*M*-connectome at the largest *N*_*F*_. A part of this graph, containing only the edges of a substantial weight, is shown in Fig. 6b. In the Supplementary Information list L-I, the names of source and target brain regions of these edges are given. The complete graph *F-excess1195* is also shown in Fig. SI-3.

It should be stressed that the excess edges observed in the F-connectome are attached to the central brain graph, the common F&M-connectome, at a large number of vertices. By considering F-excess as a *separate graph*, cf. Fig. 6b, we observe that these excess edges make nonrandom patterns and have a significant variation in weights (cf. Fig. SI-1); they involve 348 different brain areas in both hemispheres as well as the edges that connect the left and right hemispheres. The properties of the F-excess1195 subgraph are also summarised in Table 2, and the distribution of distances *P*(*d*), as well as the hyperbolicity parameters with ${\delta }_{max}=4$ are shown in Fig. 5B. Noticeably, the pattern of these extra connections in the F-connectome adds some larger cycles and 112 triangles. However, they are well embedded in the structure of the F&M-connectome, such that they do not appear as isomorphic cycles, and, consequently, do not increase the hyperbolicity parameter of the F-connectome. For comparison, we show the corresponding features of the randomised version of the F-excess1195 graph. Note that for this purpose we randomise the edges *within each hemisphere separately* while keeping the cross-hemisphere edges intact, so that the brain anatomical structure is observed. The parameters of the randomised graph are also shown in Table 2. Note that several other graph-theoretic properties, see the studies in reference^22^, also differ in female and male connectomes.

## Discussion

By analysing the HCP data provided at the Budapest connectome server, we acquired three sets of networks representing the consensus female and male connectomes at different numbers of launched fibres 20 K, 200 K, and 1000 K. In addition to the standard graph parameters, by using algebraic topology methods we discovered a latent geometry that encodes higher-order connections in these brain graphs. Our main findings are:

- *Higher-order connectivity of the common F&M-connectome*. We have shown that the human connectome, consisting of the edges that are common to both F&M connectomes, possesses a hidden structure beyond the node’s pairwise connectivity. The higher-order connections between the groups of brain regions are suitably encoded by simplexes organised into larger complex structures and quantified by structure vectors, cf. Fig. 4. Remarkably, the complexity of the human connectome increases with the number of launched fibres, reaching the simplicial complexes of the order ${q}_{max}+1=14$ at ${N}_{F}=1000\,K$. Specifically, there are six such cliques, which contain nodes in different brain modules (see Fig. SI-2 and the list L-I in Supplementary Information). We note that these simplicial complexes belong to different communities, which are anatomical mesoscopic structures of the brain graphs, cf. Fig. 6a. This architecture of connections in the brain graphs can be characterised by the tools of hyperbolic geometry. In particular, we find that they are Gromov hyperbolic graphs with small hyperbolicity constant ${\delta }_{max}=3/2$, which characterises both F- and M-connectomes at 1000 K launched fibres. Hyperbolicity varies with the network density, which is directly related to *N*_*F*_. In contrast, randomised (separately within each hemisphere) links exhibit much smaller simplexes (${q}_{max}^{rand}=3$) and increased hyperbolicity parameter that points to larger cycles. These findings indicate that the brain functional geometry consists of massive simplicial complexes as part of anatomical communities within each hemisphere as well as cycles that connect different regions inside and between the two hemispheres. Understanding the potential implications of hyperbolicity for the integration-segregation dynamics of the brain circuits remains an open question for future study.
- *Structure of the excess edges in F-connectomes*. F-connectome systematically appears to be better connected, i.e., has a more significant number of edges at every *N*_*F*_. Here, a more detailed inspection of the source-and-target brain region and the weight that identifies an edge indicates that two groups of excess edges occur: (1) The edges appearing in the F-connectome at a relatively low number of fibres which can appear in the M-connectome but only if a much larger number of fibres is launched; (2) The edges that robustly appear only in the F-connectome and have not been established in the M-connectome, including the highest available number 1000 K of fibres. From the second group, the identity of 1195 edges that first appear at 200 K in the F-excess subgraph and are not present in the common F&M-connectome at 1000 K are given in Supplementary Information. In particular, Fig. SI-3 shows the complete graph, while the list L-II contains only the edges with large weights. A comparison with the (inside the hemisphere) randomised graph has shown that these F-excess edges, considered as a separate graph, also have an organised structure involving a large number of brain regions, cf. Fig. 6b. Direct analysis and its hyperbolicity parameter suggest a geometry dominated by cycles and small simplexes.

To summarise, our study reveals how the *functional geometry* of human connectome can be expressed by higher-order connectivity, described by simplicial complexes and induced cycles. This kind of structure is built into the anatomical communities of the brain at the mesoscopic scale in both brain hemispheres. However, the precise role of these simplicial complexes for the dynamical segregation in brain functional complexity remains to be better understood. In this context, the developed methodology provides new topological measures of the consensus brain networks and quantifies the perceptive differences between connectomes. Specifically, in the studied female and male consensus connectomes, a part of connections is more natural to invoke in the female than in the male brain, where much more fibres need to be launched to identify them. Whereas the other fraction of such connections consists of edges that appear exclusively in the consensus female connectome, they have not been identified in the consensus male connectome.

It should be stressed that the considered consensus networks represent a kind of typical structures with the fixed number of vertices as 1015 brain regions while the edges are common for all 100 male and similarly for all 100 female, recorded within HCP in a representative set of (young and healthy) individuals. Note that, in each particular subject, the number of brain connections can deviate, e.g., being even considerably more abundant than in the respective consensus network. Moreover, the structure of possible connections is expected to vary with age, particular practice and with a development of diseases. Based on the brain imaging data, the methodology developed in this work would be suitable to reveal subtle differences between pairs of brains as well as changes in the brain of the same individual. Similar studies have been done with the patterns induced by the brain spontaneous fluctuations and content-related activity recorded by EEG^27,30,71^, complementing the traditional methods. The application of our methodology to these issues warants a separate study which would include a more detailed investigation of the role of orientation and the weights of the edges.

## Conclusions

Our analysis has revealed that the human connectome possesses a hyperbolic geometry and a complex structure on the scale between the node’s edges and the mesoscopic anatomical communities within the cerebral hemispheres. This structure, composed of simplicial complexes of different sizes and cycles that connect them, accurately describes the higher-order connectivity among different regions of the brain, divided into anatomical modules. Therefore, it can provide a reliable basis for understanding the functional complexity of the brain. Moreover, the female connectome appears to have a structure different from the common F&M-connectome, not only in the number of edges but also in its organisation expressed by these higher-order connections. It might be conjectured that these excess connections imply additional functionality of the female connectome, which can have evolutionary, biological, biochemical, and even social origins. These issues go beyond our mathematical analysis of brain graphs. However, we believe that our findings can motivate further studies to better understand the origin and functional consequences of the apparent gender differences in the human connectome.

## Supplementary information

Supplementary Information for Functional Geometry of Human Connectomes

## Data Availability

All data used in this work are available from the Budapest reference connectome 3.0, https://pitgroup.org/connectome/.
