# Supplementary material for: Functional Geometry of Human Connectomes
Source: Sci Rep. 2019 Aug 19;9:12060. doi: 10.1038/s41598-019-48568-5 (PMC6700117; doi:10.1038/s41598-019-48568-5)
Supplement: Supplementary file 1 — Supplementary Information for Functional Geometry of Human Connectomes [file 41598_2019_48568_MOESM1_ESM.pdf]

Supplementary Information for *Functional Geometry of Human Connectomes*

Bosiljka Tadić[a,b], Miroslav Andjelković[c], Roderick Melnik[d,e]
[a]*Department of Theoretical Physics, Jožef Stefan Institute, Jamova 39, Ljubljana, Slovenia;* [b]*Complexity Science Hub, Josefstaedter Strasse 39, Vienna, Austria;* [c] *Institute for Nuclear Sciences Vinča, University of Belgrade, 11000 Belgrade, Serbia;* [d]*MS2Discovery Interdisciplinary Research Institute, M2NeT Laboratory and Department of Mathematics, Wilfrid Laurier University, Waterloo, ON, Canada,* [e]*BCAM - Basque Center for Applied Mathematics, Alameda de Mazarredo 14, E-48009 Bilbao, Spain*

Contents

1. Fig. SI-1: Weights of edges 1

2. Tables SI-I,II: Structure vectors for the F- and M-connectomes at 1000K fibres 1

3. List L-I: Vertices forming six 14-cliques in human connectome at 1000K and Fig.SI-2 2

4. List L-II: The excess edges with large weights in the F-connectome 2

5. Fig. SI-3: The complete subgraph F-excess1195 9

1. FIG. SI-1: WEIGHTS OF EDGES

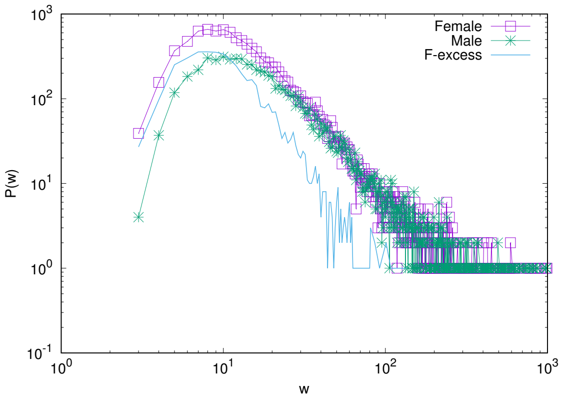

FIG. SI-1: Histogram of the weights of the edges in the F-connectome, the M-connectome and in the F-excess1195 subgraph.

2. TABLES SI-I,II: STRUCTURE VECTORS FOR THE F- AND M-CONNECTOMES AT 1000K FIBRES

Table-SI-I (structure vectors at 1000K) and Table-SI-II (some properties of SV at different N_F).

TABLE SI-I: Components of the first (FSV), second (SSV) and third (TSV) structure vectors against the topology levels q for the consensus Male and Female connectomes at 1000K fibres launched.

	Male			Female		
q	FSV	SSV	TSV	FSV	SSV	TSV
0	91	2000	0.954	45	2893	0.984
1	59	1910	0.969	33	2849	0.988
2	66	1852	0.964	33	2817	0.988
3	114	1789	0.936	62	2785	0.977
4	222	1678	0.867	91	2726	0.966
5	314	1464	0.785	118	2636	0.956
6	339	1173	0.710	199	2522	0.921
7	298	854	0.651	219	2329	0.905
8	277	587	0.528	356	2121	0.832
9	140	337	0.584	420	1782	0.764
10	110	213	0.483	353	1378	0.743
11	83	114	0.271	359	1048	0.657
12	35	39	0.102	336	712	0.528
13	6	6	0.0	183	410	0.553
14	-	-	-	126	251	0.498
15	-	-	-	70	134	0.477
16	-	-	-	49	68	0.279
17	-	-	-	19	25	0.240
18	-	-	-	8	10	0.200
19	-	-	-	4	4	0.0
20	-	-	-	1	1	0.0

TABLE SI-II: For different numbers of launched fibers N_F, some properties of the structure vectors: $Q^{\kappa}_{q=0}$–the number of graph fragments; q^{κ}_{max}—the order of the largest clique in the graph (the number of cliques of that order is given in the brackets); $\hat{Q}^{\kappa}_{q_{max}-1}$—the degree of connectivity at the level below the q_{max}, where $\kappa \in M, F$ indicating the M- or F-connectome.

N_F	$Q^M_{q=0}$	$Q^F_{q=0}$	q^M_{max}	q^F_{max}	$\hat{Q}^M_{q_{max}-1}$	$\hat{Q}^F_{q_{max}-1}$
20000	604	446	5(6)	6(8)	0.154	0.185
200000	203	109	11(3)	14(2)	0.250	0.125
1000000	91	45	13(6)	20(1)	0.103	0.00

3. LIST L-I: VERTICES FORMING SIX 14-CLIQUES IN HUMAN CONNECTOME AT 1000K AND FIG.SI-2

Names of the brain regions belonging to six largest cliques in the human connectome common to F&M-connectome. The 16 vertices in the right hemisphere and, similarly, the 16 vertices in the left hemisphere share three cliques of the order 13. Their mutual connections as directed weighted edges are shown in Fig. SI-2.

```
52 "rh.rostralmiddlefrontal_19"
53 "rh.rostralmiddlefrontal_21"
56 "rh.rostralmiddlefrontal_24"
57 "rh.rostralmiddlefrontal_10"
58 "rh.rostralmiddlefrontal_20"
60 "rh.rostralmiddlefrontal_18"
63 "rh.rostralmiddlefrontal_23"
94 "rh.superiorfrontal_11"
128 "rh.caudalmiddlefrontal_10"
129 "rh.caudalmiddlefrontal_1"
131 "rh.caudalmiddlefrontal_6"
503 "Right-Caudate"
504 "Right-Putamen"
70 "rh.rostralmiddlefrontal_2"
495 "rh.insula_1"
517 "lh.lateralorbitofrontal_10"
523 "lh.lateralorbitofrontal_1"
533 "lh.medialorbitofrontal_4"
534 "lh.medialorbitofrontal_5"
545 "lh.parstriangularis_6"
546 "lh.parstriangularis_2"
567 "lh.rostralmiddlefrontal_26"
573 "lh.rostralmiddlefrontal_23"
578 "lh.rostralmiddlefrontal_7"
580 "lh.rostralmiddlefrontal_3"
585 "lh.superiorfrontal_32"
690 "lh.rostralanteriorcingulate_3"
1009 "Left-Caudate"
1010 "Left-Putamen"
689 "lh.rostralanteriorcingulate_4"
583 "lh.rostralmiddlefrontal_1"
```

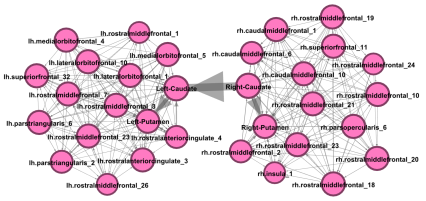

FIG. SI-2: Patterns of 239 directed weighted connections in the largest simlicial complex containing six 14-cliques in the common F&M-connectome at 1000K.

4. LIST L-II: THE EXCESS EDGES WITH LARGE WEIGHTS IN THE F-CONNECTOME

The listed edges have weights $w > 18$, i.e., in the tale of the weight distribution for F-excess, cf. Fig. SI-1).

```
203 "rh.isthmuscingulate_5" 503 "Right-Caudate"
205 "rh.postcentral_24" 237 "rh.supramarginal_11"
205 "rh.postcentral_24" 238 "rh.supramarginal_13"
205 "rh.postcentral_24" 254 "rh.supramarginal_17"
210 "rh.postcentral_11" 504 "Right-Putamen"
213 "rh.postcentral_27" 237 "rh.supramarginal_11"
213 "rh.postcentral_27" 502 "Right-Thalamus-Proper"
216 "rh.postcentral_9" 503 "Right-Caudate"
218 "rh.postcentral_30" 255 "rh.superiorparietal_13"
218 "rh.postcentral_30" 488 "rh.insula_6"
218 "rh.postcentral_30" 502 "Right-Thalamus-Proper"
223 "rh.postcentral_29" 502 "Right-Thalamus-Proper"
223 "rh.postcentral_29" 505 "Right-Pallidum"
226 "rh.postcentral_14" 502 "Right-Thalamus-Proper"
228 "rh.postcentral_21" 502 "Right-Thalamus-Proper"
228 "rh.postcentral_21" 503 "Right-Caudate"
229 "rh.postcentral_8" 237 "rh.supramarginal_11"
229 "rh.postcentral_8" 254 "rh.supramarginal_17"
229 "rh.postcentral_8" 503 "Right-Caudate"
235 "rh.supramarginal_19" 246 "rh.supramarginal_14"
235 "rh.supramarginal_19" 505 "Right-Pallidum"
238 "rh.supramarginal_13" 502 "Right-Thalamus-Proper"
```

```
241 "rh.supramarginal_8" 285 "rh.inferiorparietal_21"
244 "rh.supramarginal_9" 502 "Right-Thalamus-Proper"
244 "rh.supramarginal_9" 503 "Right-Caudate"
246 "rh.supramarginal_14" 284 "rh.inferiorparietal_13"
249 "rh.supramarginal_6" 257 "rh.superiorparietal_18"
249 "rh.supramarginal_6" 269 "rh.superiorparietal_22"
249 "rh.supramarginal_6" 285 "rh.inferiorparietal_21"
249 "rh.supramarginal_6" 327 "rh.precuneus_4"
249 "rh.supramarginal_6" 503 "Right-Caudate"
250 "rh.supramarginal_12" 284 "rh.inferiorparietal_13"
250 "rh.supramarginal_12" 328 "rh.precuneus_5"
251 "rh.supramarginal_1" 502 "Right-Thalamus-Proper"
251 "rh.supramarginal_1" 503 "Right-Caudate"
251 "rh.supramarginal_1" 504 "Right-Putamen"
252 "rh.supramarginal_7" 502 "Right-Thalamus-Proper"
253 "rh.supramarginal_5" 507 "Right-Hippocampus"
254 "rh.supramarginal_17" 257 "rh.superiorparietal_18"
254 "rh.supramarginal_17" 276 "rh.superiorparietal_11"
254 "rh.supramarginal_17" 326 "rh.precuneus_20"
255 "rh.superiorparietal_13" 276 "rh.superiorparietal_11"
255 "rh.superiorparietal_13" 315 "rh.precuneus_10"
255 "rh.superiorparietal_13" 329 "rh.precuneus_23"
255 "rh.superiorparietal_13" 505 "Right-Pallidum"
256 "rh.superiorparietal_24" 319 "rh.precuneus_9"
256 "rh.superiorparietal_24" 455 "rh.bankssts_1"
256 "rh.superiorparietal_24" 503 "Right-Caudate"
261 "rh.superiorparietal_12" 327 "rh.precuneus_4"
265 "rh.superiorparietal_17" 329 "rh.precuneus_23"
266 "rh.superiorparietal_7" 327 "rh.precuneus_4"
267 "rh.superiorparietal_20" 325 "rh.precuneus_8"
269 "rh.superiorparietal_22" 301 "rh.inferiorparietal_18"
269 "rh.superiorparietal_22" 504 "Right-Putamen"
270 "rh.superiorparietal_9" 301 "rh.inferiorparietal_18"
270 "rh.superiorparietal_9" 313 "rh.precuneus_6"
275 "rh.superiorparietal_14" 301 "rh.inferiorparietal_18"
275 "rh.superiorparietal_14" 329 "rh.precuneus_23"
275 "rh.superiorparietal_14" 504 "Right-Putamen"
276 "rh.superiorparietal_11" 290 "rh.inferiorparietal_12"
276 "rh.superiorparietal_11" 301 "rh.inferiorparietal_18"
276 "rh.superiorparietal_11" 486 "rh.insula_16"
276 "rh.superiorparietal_11" 504 "Right-Putamen"
278 "rh.superiorparietal_19" 311 "rh.precuneus_1"
279 "rh.superiorparietal_6" 311 "rh.precuneus_1"
282 "rh.superiorparietal_3" 385 "rh.lingual_6"
284 "rh.inferiorparietal_13" 299 "rh.inferiorparietal_14"
284 "rh.inferiorparietal_13" 303 "rh.inferiorparietal_20"
284 "rh.inferiorparietal_13" 311 "rh.precuneus_1"
284 "rh.inferiorparietal_13" 315 "rh.precuneus_10"
284 "rh.inferiorparietal_13" 326 "rh.precuneus_20"
284 "rh.inferiorparietal_13" 327 "rh.precuneus_4"
284 "rh.inferiorparietal_13" 385 "rh.lingual_6"
284 "rh.inferiorparietal_13" 455 "rh.bankssts_1"
284 "rh.inferiorparietal_13" 485 "rh.transversetemporal_3"
284 "rh.inferiorparietal_13" 490 "rh.insula_5"
284 "rh.inferiorparietal_13" 504 "Right-Putamen"
285 "rh.inferiorparietal_21" 289 "rh.inferiorparietal_25"
```

```
285 "rh.inferiorparietal_21" 312 "rh.precuneus_3"
285 "rh.inferiorparietal_21" 313 "rh.precuneus_6"
285 "rh.inferiorparietal_21" 316 "rh.precuneus_16"
285 "rh.inferiorparietal_21" 486 "rh.insula_16"
285 "rh.inferiorparietal_21" 504 "Right-Putamen"
290 "rh.inferiorparietal_12" 294 "rh.inferiorparietal_2"
291 "rh.inferiorparietal_8" 293 "rh.inferiorparietal_26"
291 "rh.inferiorparietal_8" 297 "rh.inferiorparietal_1"
295 "rh.inferiorparietal_17" 301 "rh.inferiorparietal_18"
295 "rh.inferiorparietal_17" 385 "rh.lingual_6"
295 "rh.inferiorparietal_17" 486 "rh.insula_16"
295 "rh.inferiorparietal_17" 490 "rh.insula_5"
295 "rh.inferiorparietal_17" 504 "Right-Putamen"
299 "rh.inferiorparietal_14" 311 "rh.precuneus_1"
299 "rh.inferiorparietal_14" 504 "Right-Putamen"
300 "rh.inferiorparietal_9" 308 "rh.inferiorparietal_15"
301 "rh.inferiorparietal_18" 326 "rh.precuneus_20"
301 "rh.inferiorparietal_18" 388 "rh.lingual_5"
301 "rh.inferiorparietal_18" 397 "rh.fusiform_15"
301 "rh.inferiorparietal_18" 407 "rh.parahippocampal_2"
301 "rh.inferiorparietal_18" 486 "rh.insula_16"
302 "rh.inferiorparietal_10" 467 "rh.superiortemporal_21"
302 "rh.inferiorparietal_10" 473 "rh.superiortemporal_11"
303 "rh.inferiorparietal_20" 341 "rh.pericalcarine_1"
303 "rh.inferiorparietal_20" 504 "Right-Putamen"
304 "rh.inferiorparietal_22" 311 "rh.precuneus_1"
308 "rh.inferiorparietal_15" 390 "rh.fusiform_2"
308 "rh.inferiorparietal_15" 486 "rh.insula_16"
309 "rh.inferiorparietal_24" 313 "rh.precuneus_6"
309 "rh.inferiorparietal_24" 342 "rh.pericalcarine_5"
313 "rh.precuneus_6" 359 "rh.lateraloccipital_3"
328 "rh.precuneus_5" 504 "Right-Putamen"
342 "rh.pericalcarine_5" 349 "rh.lateraloccipital_18"
342 "rh.pericalcarine_5" 356 "rh.lateraloccipital_16"
345 "rh.pericalcarine_2" 362 "rh.lateraloccipital_21"
356 "rh.lateraloccipital_16" 363 "rh.lateraloccipital_10"
357 "rh.lateraloccipital_23" 385 "rh.lingual_6"
358 "rh.lateraloccipital_7" 381 "rh.lingual_3"
365 "rh.lateraloccipital_4" 369 "rh.lateraloccipital_5"
366 "rh.lateraloccipital_19" 369 "rh.lateraloccipital_5"
385 "rh.lingual_6" 455 "rh.bankssts_1"
388 "rh.lingual_5" 433 "rh.middletemporal_12"
388 "rh.lingual_5" 455 "rh.bankssts_1"
396 "rh.fusiform_17" 455 "rh.bankssts_1"
401 "rh.fusiform_4" 432 "rh.inferiortemporal_13"
401 "rh.fusiform_4" 433 "rh.middletemporal_12"
410 "rh.parahippocampal_5" 502 "Right-Thalamus-Proper"
419 "rh.inferiortemporal_5" 454 "rh.bankssts_2"
425 "rh.inferiortemporal_7" 432 "rh.inferiortemporal_13"
425 "rh.inferiortemporal_7" 434 "rh.middletemporal_16"
433 "rh.middletemporal_12" 440 "rh.middletemporal_4"
433 "rh.middletemporal_12" 467 "rh.superiortemporal_21"
433 "rh.middletemporal_12" 473 "rh.superiortemporal_11"
445 "rh.middletemporal_9" 454 "rh.bankssts_2"
449 "rh.middletemporal_19" 454 "rh.bankssts_2"
450 "rh.middletemporal_2" 454 "rh.bankssts_2"
```

```
451 "rh.middletemporal_18" 467 "rh.superiortemporal_21"
451 "rh.middletemporal_18" 468 "rh.superiortemporal_24"
454 "rh.bankssts_2" 490 "rh.insula_5"
455 "rh.bankssts_1" 504 "Right-Putamen"
467 "rh.superiortemporal_21" 477 "rh.superiortemporal_8"
467 "rh.superiortemporal_21" 480 "rh.superiortemporal_20"
477 "rh.superiortemporal_8" 507 "Right-Hippocampus"
497 "rh.insula_14" 507 "Right-Hippocampus"
498 "rh.insula_3" 502 "Right-Thalamus-Proper"
500 "rh.insula_12" 507 "Right-Hippocampus"
502 "Right-Thalamus-Proper" 706 "lh.posteriorcingulate_1"
503 "Right-Caudate" 697 "lh.caudalanteriorcingulate_3"
504 "Right-Putamen" 1009 "Left-Caudate"
524 "lh.lateralorbitofrontal_15" 1009 "Left-Caudate"
545 "lh.parstriangularis_6" 572 "lh.rostralmiddlefrontal_21"
545 "lh.parstriangularis_6" 584 "lh.superiorfrontal_11"
545 "lh.parstriangularis_6" 586 "lh.superiorfrontal_8"
548 "lh.parsopercularis_6" 1013 "Left-Hippocampus"
552 "lh.parsopercularis_8" 560 "lh.rostralmiddlefrontal_13"
552 "lh.parsopercularis_8" 693 "lh.caudalanteriorcingulate_5"
552 "lh.parsopercularis_8" 1011 "Left-Pallidum"
555 "lh.parsopercularis_3" 1009 "Left-Caudate"
558 "lh.rostralmiddlefrontal_17" 629 "lh.caudalmiddlefrontal_9"
562 "lh.rostralmiddlefrontal_19" 637 "lh.caudalmiddlefrontal_4"
566 "lh.rostralmiddlefrontal_4" 571 "lh.rostralmiddlefrontal_10"
566 "lh.rostralmiddlefrontal_4" 601 "lh.superiorfrontal_21"
566 "lh.rostralmiddlefrontal_4" 637 "lh.caudalmiddlefrontal_4"
567 "lh.rostralmiddlefrontal_26" 590 "lh.superiorfrontal_35"
591 "lh.superiorfrontal_42" 595 "lh.superiorfrontal_9"
591 "lh.superiorfrontal_42" 615 "lh.superiorfrontal_18"
596 "lh.superiorfrontal_1" 613 "lh.superiorfrontal_7"
596 "lh.superiorfrontal_1" 616 "lh.superiorfrontal_34"
597 "lh.superiorfrontal_38" 686 "lh.paracentral_5"
598 "lh.superiorfrontal_45" 615 "lh.superiorfrontal_18"
601 "lh.superiorfrontal_21" 695 "lh.caudalanteriorcingulate_1"
602 "lh.superiorfrontal_29" 694 "lh.caudalanteriorcingulate_4"
603 "lh.superiorfrontal_44" 651 "lh.precentral_21"
603 "lh.superiorfrontal_44" 687 "lh.paracentral_11"
603 "lh.superiorfrontal_44" 696 "lh.caudalanteriorcingulate_2"
604 "lh.superiorfrontal_19" 1009 "Left-Caudate"
605 "lh.superiorfrontal_30" 607 "lh.superiorfrontal_22"
607 "lh.superiorfrontal_22" 611 "lh.superiorfrontal_40"
607 "lh.superiorfrontal_22" 686 "lh.paracentral_5"
608 "lh.superiorfrontal_27" 644 "lh.precentral_12"
609 "lh.superiorfrontal_20" 686 "lh.paracentral_5"
612 "lh.superiorfrontal_36" 695 "lh.caudalanteriorcingulate_1"
612 "lh.superiorfrontal_36" 1009 "Left-Caudate"
613 "lh.superiorfrontal_7" 632 "lh.caudalmiddlefrontal_13"
613 "lh.superiorfrontal_7" 697 "lh.caudalanteriorcingulate_3"
616 "lh.superiorfrontal_34" 1009 "Left-Caudate"
618 "lh.superiorfrontal_3" 625 "lh.superiorfrontal_6"
618 "lh.superiorfrontal_3" 685 "lh.paracentral_6"
622 "lh.superiorfrontal_15" 1009 "Left-Caudate"
623 "lh.superiorfrontal_43" 683 "lh.paracentral_3"
623 "lh.superiorfrontal_43" 687 "lh.paracentral_11"
624 "lh.superiorfrontal_26" 662 "lh.precentral_25"
```

```
624 "lh.superiorfrontal_26" 686 "lh.paracentral_5"
628 "lh.superiorfrontal_41" 683 "lh.paracentral_3"
629 "lh.caudalmiddlefrontal_9" 651 "lh.precentral_21"
629 "lh.caudalmiddlefrontal_9" 657 "lh.precentral_4"
629 "lh.caudalmiddlefrontal_9" 661 "lh.precentral_6"
632 "lh.caudalmiddlefrontal_13" 694 "lh.caudalanteriorcingulate_4"
632 "lh.caudalmiddlefrontal_13" 698 "lh.posteriorcingulate_5"
632 "lh.caudalmiddlefrontal_13" 699 "lh.posteriorcingulate_4"
632 "lh.caudalmiddlefrontal_13" 1013 "Left-Hippocampus"
633 "lh.caudalmiddlefrontal_5" 731 "lh.postcentral_31"
633 "lh.caudalmiddlefrontal_5" 769 "lh.superiorparietal_27"
633 "lh.caudalmiddlefrontal_5" 770 "lh.superiorparietal_25"
636 "lh.caudalmiddlefrontal_11" 641 "lh.caudalmiddlefrontal_10"
636 "lh.caudalmiddlefrontal_11" 1009 "Left-Caudate"
637 "lh.caudalmiddlefrontal_4" 658 "lh.precentral_13"
637 "lh.caudalmiddlefrontal_4" 662 "lh.precentral_25"
641 "lh.caudalmiddlefrontal_10" 697 "lh.caudalanteriorcingulate_3"
645 "lh.precentral_17" 686 "lh.paracentral_5"
647 "lh.precentral_33" 661 "lh.precentral_6"
647 "lh.precentral_33" 731 "lh.postcentral_31"
647 "lh.precentral_33" 755 "lh.supramarginal_17"
647 "lh.precentral_33" 757 "lh.supramarginal_16"
650 "lh.precentral_26" 662 "lh.precentral_25"
650 "lh.precentral_26" 681 "lh.paracentral_10"
651 "lh.precentral_21" 666 "lh.precentral_11"
651 "lh.precentral_21" 668 "lh.precentral_8"
651 "lh.precentral_21" 669 "lh.precentral_15"
651 "lh.precentral_21" 720 "lh.postcentral_18"
651 "lh.precentral_21" 768 "lh.superiorparietal_20"
651 "lh.precentral_21" 769 "lh.superiorparietal_27"
651 "lh.precentral_21" 770 "lh.superiorparietal_25"
651 "lh.precentral_21" 820 "lh.precuneus_10"
652 "lh.precentral_10" 1008 "Left-Thalamus-Proper"
654 "lh.precentral_16" 770 "lh.superiorparietal_25"
654 "lh.precentral_16" 771 "lh.superiorparietal_13"
657 "lh.precentral_4" 769 "lh.superiorparietal_27"
657 "lh.precentral_4" 770 "lh.superiorparietal_25"
664 "lh.precentral_20" 1010 "Left-Putamen"
665 "lh.precentral_29" 1008 "Left-Thalamus-Proper"
669 "lh.precentral_15" 699 "lh.posteriorcingulate_4"
669 "lh.precentral_15" 758 "lh.supramarginal_15"
669 "lh.precentral_15" 770 "lh.superiorparietal_25"
669 "lh.precentral_15" 1013 "Left-Hippocampus"
675 "lh.precentral_28" 757 "lh.supramarginal_16"
676 "lh.precentral_23" 756 "lh.supramarginal_4"
676 "lh.precentral_23" 758 "lh.supramarginal_15"
687 "lh.paracentral_11" 757 "lh.supramarginal_16"
691 "lh.rostralanteriorcingulate_2" 698 "lh.posteriorcingulate_5"
691 "lh.rostralanteriorcingulate_2" 1005 "lh.insula_7"
692 "lh.rostralanteriorcingulate_5" 1005 "lh.insula_7"
697 "lh.caudalanteriorcingulate_3" 705 "lh.posteriorcingulate_7"
697 "lh.caudalanteriorcingulate_3" 706 "lh.posteriorcingulate_1"
697 "lh.caudalanteriorcingulate_3" 709 "lh.isthmuscingulate_7"
703 "lh.posteriorcingulate_6" 755 "lh.supramarginal_17"
706 "lh.posteriorcingulate_1" 1013 "Left-Hippocampus"
707 "lh.isthmuscingulate_4" 712 "lh.isthmuscingulate_2"
```

```
707 "lh.isthmuscingulate_4" 769 "lh.superiorparietal_27"
708 "lh.isthmuscingulate_6" 713 "lh.isthmuscingulate_3"
708 "lh.isthmuscingulate_6" 769 "lh.superiorparietal_27"
708 "lh.isthmuscingulate_6" 770 "lh.superiorparietal_25"
709 "lh.isthmuscingulate_7" 768 "lh.superiorparietal_20"
709 "lh.isthmuscingulate_7" 776 "lh.superiorparietal_19"
709 "lh.isthmuscingulate_7" 836 "lh.precuneus_9"
710 "lh.isthmuscingulate_1" 770 "lh.superiorparietal_25"
711 "lh.isthmuscingulate_5" 1009 "Left-Caudate"
712 "lh.isthmuscingulate_2" 1009 "Left-Caudate"
718 "lh.postcentral_14" 1008 "Left-Thalamus-Proper"
720 "lh.postcentral_18" 991 "lh.insula_5"
720 "lh.postcentral_18" 1008 "Left-Thalamus-Proper"
720 "lh.postcentral_18" 1009 "Left-Caudate"
726 "lh.postcentral_30" 992 "lh.insula_2"
726 "lh.postcentral_30" 1008 "Left-Thalamus-Proper"
730 "lh.postcentral_8" 991 "lh.insula_5"
730 "lh.postcentral_8" 1008 "Left-Thalamus-Proper"
731 "lh.postcentral_31" 758 "lh.supramarginal_15"
731 "lh.postcentral_31" 770 "lh.superiorparietal_25"
738 "lh.postcentral_10" 1008 "Left-Thalamus-Proper"
738 "lh.postcentral_10" 1009 "Left-Caudate"
738 "lh.postcentral_10" 1011 "Left-Pallidum"
742 "lh.postcentral_19" 755 "lh.supramarginal_17"
746 "lh.supramarginal_7" 1008 "Left-Thalamus-Proper"
747 "lh.supramarginal_5" 1008 "Left-Thalamus-Proper"
749 "lh.supramarginal_13" 1009 "Left-Caudate"
750 "lh.supramarginal_2" 768 "lh.superiorparietal_20"
750 "lh.supramarginal_2" 774 "lh.superiorparietal_15"
750 "lh.supramarginal_2" 779 "lh.superiorparietal_16"
750 "lh.supramarginal_2" 1003 "lh.insula_15"
750 "lh.supramarginal_2" 1004 "lh.insula_4"
751 "lh.supramarginal_3" 1009 "Left-Caudate"
755 "lh.supramarginal_17" 1011 "Left-Pallidum"
756 "lh.supramarginal_4" 1008 "Left-Thalamus-Proper"
757 "lh.supramarginal_16" 997 "lh.insula_14"
757 "lh.supramarginal_16" 1011 "Left-Pallidum"
758 "lh.supramarginal_15" 1010 "Left-Putamen"
767 "lh.superiorparietal_24" 1011 "Left-Pallidum"
768 "lh.superiorparietal_20" 1008 "Left-Thalamus-Proper"
768 "lh.superiorparietal_20" 1009 "Left-Caudate"
769 "lh.superiorparietal_27" 832 "lh.precuneus_6"
769 "lh.superiorparietal_27" 1011 "Left-Pallidum"
770 "lh.superiorparietal_25" 776 "lh.superiorparietal_19"
770 "lh.superiorparietal_25" 796 "lh.inferiorparietal_16"
770 "lh.superiorparietal_25" 993 "lh.insula_16"
770 "lh.superiorparietal_25" 1011 "Left-Pallidum"
771 "lh.superiorparietal_13" 831 "lh.precuneus_22"
771 "lh.superiorparietal_13" 965 "lh.superiortemporal_24"
771 "lh.superiorparietal_13" 1008 "Left-Thalamus-Proper"
775 "lh.superiorparietal_12" 821 "lh.precuneus_8"
775 "lh.superiorparietal_12" 822 "lh.precuneus_3"
779 "lh.superiorparietal_16" 965 "lh.superiortemporal_24"
779 "lh.superiorparietal_16" 1010 "Left-Putamen"
780 "lh.superiorparietal_11" 965 "lh.superiortemporal_24"
780 "lh.superiorparietal_11" 1010 "Left-Putamen"
```

```
781 "lh.superiorparietal_18" 820 "lh.precuneus_10"
784 "lh.superiorparietal_4" 832 "lh.precuneus_6"
785 "lh.superiorparietal_6" 1010 "Left-Putamen"
786 "lh.superiorparietal_2" 808 "lh.inferiorparietal_21"
786 "lh.superiorparietal_2" 892 "lh.lingual_7"
786 "lh.superiorparietal_2" 965 "lh.superiortemporal_24"
786 "lh.superiorparietal_2" 993 "lh.insula_16"
786 "lh.superiorparietal_2" 1010 "Left-Putamen"
790 "lh.superiorparietal_9" 849 "lh.pericalcarine_2"
794 "lh.superiorparietal_29" 849 "lh.pericalcarine_2"
796 "lh.inferiorparietal_16" 851 "lh.pericalcarine_3"
796 "lh.inferiorparietal_16" 869 "lh.lateraloccipital_21"
796 "lh.inferiorparietal_16" 870 "lh.lateraloccipital_23"
796 "lh.inferiorparietal_16" 871 "lh.lateraloccipital_5"
796 "lh.inferiorparietal_16" 875 "lh.lateraloccipital_8"
796 "lh.inferiorparietal_16" 893 "lh.fusiform_12"
796 "lh.inferiorparietal_16" 894 "lh.fusiform_7"
796 "lh.inferiorparietal_16" 1010 "Left-Putamen"
801 "lh.inferiorparietal_15" 807 "lh.inferiorparietal_17"
801 "lh.inferiorparietal_15" 888 "lh.lingual_2"
801 "lh.inferiorparietal_15" 993 "lh.insula_16"
801 "lh.inferiorparietal_15" 1010 "Left-Putamen"
801 "lh.inferiorparietal_15" 1013 "Left-Hippocampus"
805 "lh.inferiorparietal_2" 814 "lh.inferiorparietal_14"
805 "lh.inferiorparietal_2" 993 "lh.insula_16"
805 "lh.inferiorparietal_2" 1010 "Left-Putamen"
806 "lh.inferiorparietal_18" 815 "lh.inferiorparietal_1"
806 "lh.inferiorparietal_18" 889 "lh.lingual_15"
806 "lh.inferiorparietal_18" 994 "lh.insula_12"
807 "lh.inferiorparietal_17" 888 "lh.lingual_2"
807 "lh.inferiorparietal_17" 895 "lh.fusiform_17"
807 "lh.inferiorparietal_17" 994 "lh.insula_12"
808 "lh.inferiorparietal_21" 815 "lh.inferiorparietal_1"
808 "lh.inferiorparietal_21" 989 "lh.transversetemporal_3"
808 "lh.inferiorparietal_21" 990 "lh.transversetemporal_2"
808 "lh.inferiorparietal_21" 993 "lh.insula_16"
808 "lh.inferiorparietal_21" 1008 "Left-Thalamus-Proper"
808 "lh.inferiorparietal_21" 1010 "Left-Putamen"
809 "lh.inferiorparietal_12" 815 "lh.inferiorparietal_1"
809 "lh.inferiorparietal_12" 993 "lh.insula_16"
809 "lh.inferiorparietal_12" 1010 "Left-Putamen"
815 "lh.inferiorparietal_1" 965 "lh.superiortemporal_24"
815 "lh.inferiorparietal_1" 1010 "Left-Putamen"
816 "lh.inferiorparietal_20" 833 "lh.precuneus_11"
816 "lh.inferiorparietal_20" 838 "lh.precuneus_18"
816 "lh.inferiorparietal_20" 852 "lh.pericalcarine_6"
816 "lh.inferiorparietal_20" 888 "lh.lingual_2"
816 "lh.inferiorparietal_20" 892 "lh.lingual_7"
816 "lh.inferiorparietal_20" 993 "lh.insula_16"
816 "lh.inferiorparietal_20" 1008 "Left-Thalamus-Proper"
816 "lh.inferiorparietal_20" 1010 "Left-Putamen"
837 "lh.precuneus_15" 869 "lh.lateraloccipital_21"
848 "lh.pericalcarine_4" 862 "lh.lateraloccipital_12"
849 "lh.pericalcarine_2" 855 "lh.lateraloccipital_10"
850 "lh.pericalcarine_5" 863 "lh.lateraloccipital_14"
852 "lh.pericalcarine_6" 858 "lh.lateraloccipital_4"
```

```
869 "lh.lateraloccipital_21" 875 "lh.lateraloccipital_8"
870 "lh.lateraloccipital_23" 889 "lh.lingual_15"
870 "lh.lateraloccipital_23" 915 "lh.parahippocampal_5"
872 "lh.lateraloccipital_17" 884 "lh.lingual_6"
888 "lh.lingual_2" 959 "lh.bankssts_4"
892 "lh.lingual_7" 965 "lh.superiortemporal_24"
894 "lh.fusiform_7" 959 "lh.bankssts_4"
901 "lh.fusiform_11" 958 "lh.bankssts_6"
903 "lh.fusiform_16" 945 "lh.middletemporal_13"
904 "lh.fusiform_2" 957 "lh.bankssts_2"
906 "lh.fusiform_8" 958 "lh.bankssts_6"
909 "lh.fusiform_13" 957 "lh.bankssts_2"
914 "lh.parahippocampal_4" 959 "lh.bankssts_4"
915 "lh.parahippocampal_5" 938 "lh.inferiortemporal_7"
915 "lh.parahippocampal_5" 959 "lh.bankssts_4"
923 "lh.inferiortemporal_15" 957 "lh.bankssts_2"
923 "lh.inferiortemporal_15" 959 "lh.bankssts_4"
927 "lh.inferiortemporal_5" 958 "lh.bankssts_6"
928 "lh.inferiortemporal_8" 958 "lh.bankssts_6"
934 "lh.inferiortemporal_3" 939 "lh.middletemporal_6"
952 "lh.middletemporal_2" 957 "lh.bankssts_2"
953 "lh.middletemporal_4" 1013 "Left-Hippocampus"
957 "lh.bankssts_2" 994 "lh.insula_12"
958 "lh.bankssts_6" 1013 "Left-Hippocampus"
959 "lh.bankssts_4" 989 "lh.transversetemporal_3"
959 "lh.bankssts_4" 1010 "Left-Putamen"
970 "lh.superiortemporal_25" 989 "lh.transversetemporal_3"
981 "lh.superiortemporal_10" 1013 "Left-Hippocampus"
1007 "lh.insula_17" 1013 "Left-Hippocampus"
```

5. FIG. SI-3: THE COMPLETE SUBGRAPH F-EXCESS1195

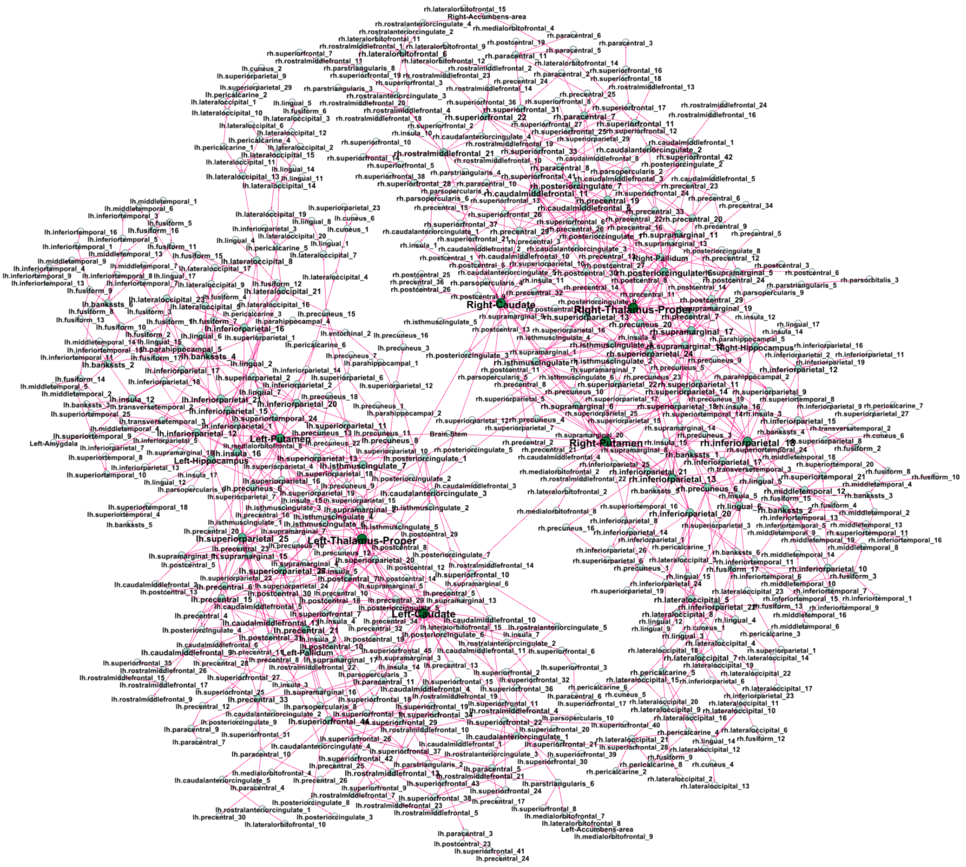

FIG. SI-3: The structure of the subgraph F-excess1195 consisting of the 1195 excess edges in the F-connectome.
